# SeedSortNet: a rapid and highly effificient lightweight CNN based on visual attention for seed sorting

**DOI:** 10.7717/peerj-cs.639

**Published:** 2021-08-05

**Authors:** Chunlei Li, Huanyu Li, Zhoufeng Liu, Bicao Li, Yun Huang

**Affiliations:** 1 School of Electrical and Information Engineering, Zhongyuan University of Technology, Zhengzhou, Henan, China; 2 Xiamen Vision+ Technology Co. Ltd, Xiamen, Fujian, China

**Keywords:** Seed sorting, Computer vision, Lightweight CNN, Attention mechanism

## Abstract

Seed purity directly affects the quality of seed breeding and subsequent processing products. Seed sorting based on machine vision provides an effective solution to this problem. The deep learning technology, particularly convolutional neural networks (CNNs), have exhibited impressive performance in image recognition and classification, and have been proven applicable in seed sorting. However the huge computational complexity and massive storage requirements make it a great challenge to deploy them in real-time applications, especially on devices with limited resources. In this study, a rapid and highly efficient lightweight CNN based on visual attention, namely SeedSortNet, is proposed for seed sorting. First, a dual-branch lightweight feature extraction module Shield-block is elaborately designed by performing identity mapping, spatial transformation at higher dimensions and different receptive field modeling, and thus it can alleviate information loss and effectively characterize the multi-scale feature while utilizing fewer parameters and lower computational complexity. In the down-sampling layer, the traditional MaxPool is replaced as MaxBlurPool to improve the shift-invariant of the network. Also, an extremely lightweight sub-feature space attention module (SFSAM) is presented to selectively emphasize fine-grained features and suppress the interference of complex backgrounds. Experimental results show that SeedSortNet achieves the accuracy rates of 97.33% and 99.56% on the maize seed dataset and sunflower seed dataset, respectively, and outperforms the mainstream lightweight networks (MobileNetv2, ShuffleNetv2, etc.) at similar computational costs, with only 0.400M parameters (vs. 4.06M, 5.40M).

## Introduction

Seed purity directly affects the quality of seed breeding and subsequent processing products. For example, in the process of seed harvest and storage, the impurities or hybrids may be mixed in the normal seed, which results in the economic losses to agricultural production and processing. Therefore, it is crucial to sort impurities and hybrids to ensure that the seed purity meet the market criteria. However, the traditional manual sorting methods are laborious and time-consuming, and hence less efficient. With the evolution of the technology, there has been a tremendous development in the field of machine vision (Rehman et al., 2019; Wu et al., 2020) and robot control technology (Liu, Yu & Cang, 2019; Liu et al., 2020; Han et al., 2020b). The automatic sorting methods (Li et al., 2019) based on the above technologies provide a promising solution.

Traditional automatic seed sorting methods adopt hand-crafted features for image characterization, such as color, shape, texture, and wavelet features or their combinations (Liu et al., 2015; HemaChitra & Suguna, 2018; Li et al., 2019). Then, the effective classifiers are employed to realize seed recognition such as linear discriminant analysis (LDA) (Choudhary, Paliwal & Jayas, 2008), support vector machine (SVM) (Altuntas et al., 2018), decision tree (DT) (Kayacan, Sofu & Cetisli, 2016), least square (LS) (Mebatsion, Paliwal & Jayas, 2013) and artificial neural network (ANN) (Liu et al., 2015). However, these methods are designed for a specific kind of seed and lack self-adaptivity. In the last three years, mainly due to the advances of deep learning, more concretely convolutional neural networks (CNNs), the quality of image classification (Krizhevsky, Sutskever & Hinton, 2012; Han et al., 2018), object detection (Ren et al., 2015; Sun et al., 2019; Bochkovskiy, Wang & Liao, 2020) and semantic segmentation (Chen et al., 2014) has been progressing at a dramatic pace. Recently, some researchers also adopted deep learning technology in crop identification tasks and achieved good performance (Ni et al., 2019; Kurtulmus, 2021).

The crop recognition and classification methods, especially for seed sorting, should be deployed on a fast and stable embedded system due to the requirement of higher processing speed. However, the performance of these methods often depends on a deeper, wider network structure, thus it suffers from huge computational complexity and massive storage requirements (Han et al., 2018). Therefore, the deep CNN model should be compressed and streamlined while maintaining high recognition accuracy. Recently, some lightweight and efficient CNN models have been designed, such as MobileNet (Howard et al., 2017), MobileNetv2 (Sandler et al., 2018), ShuffleNet (Zhang et al., 2018) and ShuffleNetv2 (Ma et al., 2018) for the real-time detection and recognition tasks. However, due to the lower discrimination of different types of seed, the feature extraction ability of these models is insufficient, thus leads to low recognition accuracy.

In this paper, a lightweight CNN based on visual attention for seed sorting is proposed. A dual-branch lightweight feature extraction module (i.e., Shield-block) is designed to improve the feature characterization ability while utilizing fewer parameters and lower computational complexity, and the traditional MaxPool is replaced as MaxBlurPool (Zhang, 2019) to improve the shift-invariant of the network in the down-sampling layer. In addition, an extremely lightweight sub-feature space attention module (SFSAM) is proposed as the basic unit of the built CNN model to selectively emphasize fine-grained features and suppress the interference from complex backgrounds. Overall, ours contributions are three-fold as follows:

• We designed a dual-branch lightweight feature extraction module (i.e, Shield-block) to alleviate information loss and effectively characterize the multi-scale feature while utilizing fewer parameters and lower computational complexity.
• We proposed an extremely lightweight sub-feature space attention module, which divides the feature maps into different subspaces and infers different attention maps for each subspace. To selectively emphasize fine-grained features, and suppress the interference of complex backgrounds.
• Experiments are conducted on the maize seed dataset and sunflower seed dataset, and the results show that SeedSortNet achieves higher accuracy compared with the mainstream lightweight networks (MobileNetv2, ShuffleNetv2, etc.) at the similar computational cost, even outperforms the deeper and wider networks, such as VGG (Simonyan & Zisserman, 2014), GoogleNet (Szegedy et al., 2015), and ResNet (He et al., 2016).

The remainder of the paper is organized as follows. In ‘Related Work’, we summarize some related work on seed sorting and lightweight model design. ‘Proposed Method’ introduces the technical details of the proposed method and network architecture. In ‘Experiments’, we carry out a series of comparative experiments on maize and sunflower seed datasets and the experimental results are analyzed. Finally, we conclude in ‘Discussion’.

## Related work

In the following, we review the existing crop identification methods and related technologies, such as CNN model compression and lightweight model design.

### Crop identification

Agricultural product assessment and recognition based on machine vision technology have been a research focus in agricultural applications, which is widely used in the detection and sorting of agricultural products such as wheat, corn, fruits, and the identification of plant diseases and insect pests.

 Liu et al. (2015) proposed a novel soybean seed sorting based on neural network. Eight shape features, three-color features, and three texture features are extracted to characterize the soybean seed, and BP neural network is used as the classification model to recognize the different defects. Experiments are conducted on the collected image set which includes 857 images of soybean seeds with insect damage, mildew, and other defects, and the results achieve an average recognition accuracy of 97.25%.  Huang (2012) proposed a neural network-based quality evaluation and classification method for areca nuts. The axis length, secondary axis length, axis number, area, perimeter, compactness, and the average gray level are used as the feature, and a back-propagation neural network classifier is employed to sort the quality of the areca nuts. Aznan et al. (2016) adopted machine vision methods to discriminate the variety of cultivated rice seed, namely M263. They firstly extracted different morphological features and then adopted a stepwise discriminant function analysis (DFA) to classify different types of rice. The classification accuracy for testing and training sets is 96% and 95.8%, respectively. HemaChitra & Suguna (2018) presented a novel sorting method of Indian pulse seeds based on image analysis techniques. In this method, they extracted the colors, shapes, and texture features, and adopted SVM for classification. The accuracy of their method can reach 98.9% accuracy. Li et al. (2019) designed a system to distinguish different damaged types of corn. An image database including normal corn and six different damaged corns is constructed. The features such as color and shape are extracted, then the maximum likelihood classifier is leveraged to discriminate these corns. Experiment results show that the classification accuracy is above 74% for all the classes. However, these methods adopt the handcraft features designed for the specific crops and the traditional classifier for sorting, and suffer from poor adaptability and low accuracy.

Due to the excellent feature representation ability, the deep learning models represented by CNN have achieved good performance in image classification, object detection, and semantic segmentation, and have also been successfully applied in plant disease detection and crop type classification. Sladojevic et al. (2016) proposed a CNN-based system to identify 13 types of plant diseases out of healthy leaves. The performance of this approach exhibited a top-1 success of 96.3%. Veeramani, Raymond & Chanda (2018) studied the effect of the number of convolution kernels in the two layers CNN on the recognition performance of haploid and diploid corn seeds. Veeramani, Raymond & Chanda (2018) adopted VGG19 and GoogleNet to classify corn seed defects and analyzed the influence of the two networks with different depths on the recognition performance. Dolata & Reiner (2018) proposed a method for the classification of barley varieties based on CNN, which is based on two separate convolutional layers to analyze dorsal and ventral sides, respectively. The network is trained on a small sample set of 200-500 cases in 8 categories, and the classification accuracy reaches 97%. Kurtulmuş (2021) adopted AlexNet (Krizhevsky, Sutskever & Hinton, 2012), GoogleNet, and ResNet to identify sunflower seed varieties, and then they were also evaluated in terms of both accuracy and training time, GoogleNet obtained the highest classification accuracy (95%).

The CNN-based crop identification method can achieve the better recognition rate and has higher self-adaptivity. However, the performance of the deep learning method depends on the depth and width of the model, the researchers often boost the depth and width of the model to improve the performance of the detection and recognition system. But this strategy results in slow speed and difficult deployment in industrial applications.

### Model lightweight

For the specific application of crop seed sorting, due to the extremely fast production speed, it is necessary to develop the lightweight CNN model while maintaining a higher recognition accuracy. To trade off the model size and performance for deep neural network architectures has been an active research area, the related technologies include model compression, lightweight network design, etc (Liang et al., 2021).

#### Model compression

Model compression aims at generating the small network models from the trained large network models while keeping the performance. The typical techniques include pruning, quantization, and knowledge distillation. Pruning technology is based on the assumption that many parameters in the deep neural network are redundant, then the weights (Guo, Yao & Chen, 2016; Aghasi et al., 2016; Liu et al., 2018) or filters (Li et al., 2016; Liu et al., 2017; Lin et al., 2020) with low correlation can be removed to make the network structure sparse. Quantization methods aim to deploy the CNN model on the terminal hardware and encode the weights and activations using 8-bit integers (INT8) without incurring a significant loss in accuracy. Some other quantization methods even adopt INT4 or lower, such as binary quantization (Courbariaux et al., 2016) and ternary quantization (Mellempudi et al., 2017) to reduce the model size. Knowledge distillation is firstly proposed by Bucilu, Caruana & Niculescu-Mizil (2006) and generalized by Hinton, Vinyals & Dean (2015) and can generate a small student network by learning the behavior of a large teacher network. Cho & Hariharan (2019) empirically analyzed in detail the efficacy of knowledge distillation. However, compared with the original network, model compression is difficult to achieve better performance. The compression size is too large, which will lead to significant decrease of performance.

#### Lightweight network design

Lightweight network design refers to the redesign of the network structure based on the existing CNN model to reduce the parameters and the computational complexity. Lin, Chen & Yan (2013) proposed a Network-In-Network architecture, which used 1×1 convolution to increase network performance while maintaining a lower computational complexity. SqueezeNet (Iandola et al., 2016) is a lightweight network structure based on 1×1 convolution. The squeeze and expand module proposed by this model can effectively reduce the parameters while ensuring recognition accuracy. The recognition accuracy of the proposed method can be up to 57.55%, and it is similar with the AlexNet with the model size of 50×smaller. Google developed two efficient architectures denoted as MobileNet (Howard et al., 2017) and MobileNetv2 (Sandler et al., 2018) in 2017 and 2018, respectively. MobileNet proposed depthwise separable convolutions to reduce the computational complexity and achieved the state-of-art accuracy with low latency. Thereafter, the linear bottleneck with inverted residual structure is proposed in MobileNetV2 to construct a more efficient architecture. ShuffleNet (Zhang et al., 2018) proposed the pointwise group convolution and channel shuffle operations to improve the recognition accuracy while reducing latency. Combining the advantage of MobileNet and ShuffleNet, Ma et al. (2018) proposed ShuffleNetV2, which improves group convolution by channel split and used channel shuffle for the split channel as well. Wang & Yu (2020) proposed the Tied Block Convolution (TBC) which shares the same thinner filters over equal blocks of channels and produces multiple responses with a single filter, to design a lightweight model. GhostNet (Han et al., 2020a) applied a series of linear transformations to generate many Ghost feature maps, and it can characterize the required information from the original features at a small cost, which effectively reduces calculation and parameters. However, due to the low discrimination of crop seeds, the recognition accuracy will be significantly reduced when the existing lightweight models are directly applied to the seed sorting. Therefore, a rapid and highly efficient lightweight CNN model should be developed based on the characteristics of crop seeds while keeping the accuracy.

## Proposed Method

**Figure 1:**
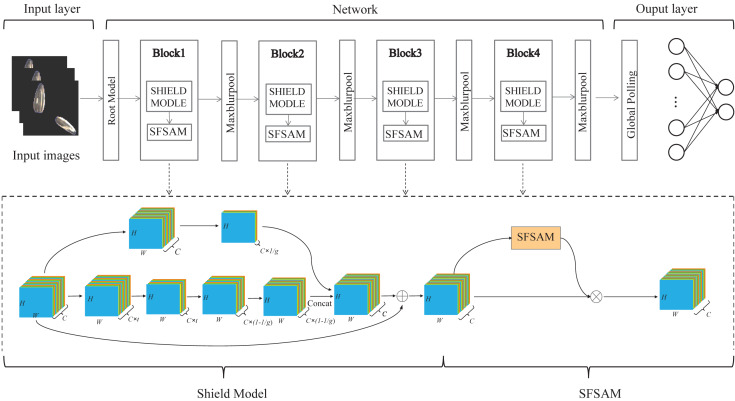
The flowchart of the proposed SeedSortNet model.

Seed sorting based on deep learning is a promising method for seed breeding and subsequent processing products. In this paper, we proposed a rapid and efficient lightweight CNN model with a dual-branch network structure based on visual attention for seed sorting, denoted as SeedSortNet. It is an efficient and lightweight end-to-end recognition framework, which is mainly composed of sequential cascade layers and basic blocks, and the overall structure of the model is shown in Fig. 1. First, a dual-branch lightweight feature extraction module, namely Shield-block, is designed for effective feature extraction. Then, the traditional convolution is replaced by depthwise convolution and pointwise convolution to achieve the trade-off between classification accuracy and efficiency. Moreover, in the down-sampling layer, MaxPool is substituted as MaxBlurPool to improve the shift-invariant of the network. Finally, we propose an extremely lightweight sub-feature space attention module to selectively emphasize fine-grained features and suppress the interference of complex backgrounds. And the proposed method is specifically described as follows.

### Network construction

Due to the required higher processing speed and recognition accuracy, the representative deep neural network model (eg, ResNet, VGG, GoogLeNet, etc.) cannot efficiently tackle with the seed sorting task because of the lower efficiency and insufficient feature extraction ability. To address these issues, we construct a novel lightweight and efficient network which consists of Root-model, Shield-block, and a novel down-sampling module.

**Figure 2:**
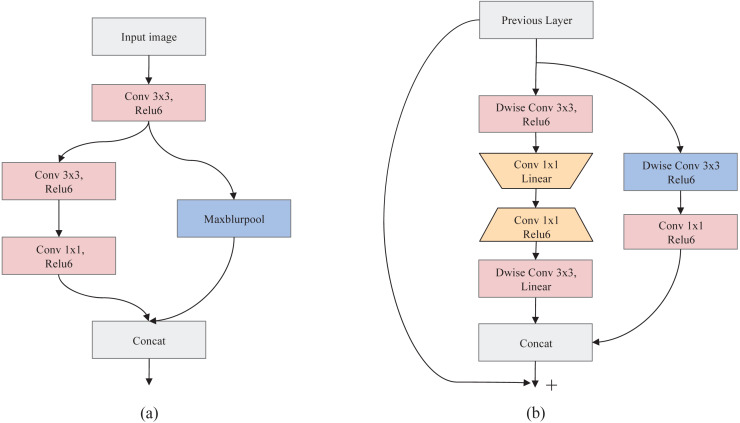
The schema of Root-model (A) and Shield-block (B).

***A.Root-model***. To effectively improve the feature representation ability while reducing calculation, a dual-branch structure, namely Root-model (Fig. 2A), is designed as the first stage of SeedSortNet. First, the sixteen 3×3 filters are utilized to extract the shallow feature information (such as texture, shape, color, etc.) of the test image. Then, in one branch, the MaxBlurPool which is a non-overlapping 2×2 window is designed for reducing the aliasing effect and improving the shift-invariant of the network. In another branch, we firstly use 3×3 filters with the stride of 2 to convolute the input features and then adopt 1×1 filters to reduce the output dimension of the branch. Finally, the features generated by the two branches are concatenated together as the input of the next layer.

***B.Shield-block***. The inverted residual block (Sandler et al., 2018) which shifts the identity mapping from high-dimensional representations to low-dimensional ones (i.e., the bottlenecks), has been successfully applied in the design of lightweight networks. However, the connection of identity mapping between thin bottlenecks would inevitably lead to information loss since the residual representations are compressed (Daquan et al., 2020). In addition, this connection would also weaken the propagation capability of gradients across layers due to gradient confusion arising from the narrowed feature dimensions, and hence affect the training convergence and model performance (Sankararaman et al., 2020). To address these issues, we propose a dual-branch feature extraction module by improving the inverted residual block in this article (shown in Fig. 2B).

In the main branch, two 3×3 depthwise convolution layers are utilized to encode richer spatial information to generate a more expressive representation. Then we adopt two pointwise convolutional layers between two 3×3 depthwise convolutional layers, the first point convolution layer reduces the feature channel dimension and the latter increases its dimension, to encode the cross-channel information of the feature maps and reduce the computational complexity. Also, the linear activation function is adopted after the first pointwise convolutional layer and the last depthwise convolutional layer, which can prevent the feature values from being zeroed and hence reduce information loss.

For the other branch, a 3×3 depthwise separable convolution is designed to acquire the spatial representation of different receptive fields, and thus improve the feature representation ability. In the end, the concatenation of the two branches and its shortcut connection with the input feature are combined as the final output.

In the following, we present the detailed data processing operator of Shield-block, and it is shown in Table 1, where *H*, *W* and *C* represents the height, width, and channel number of the feature map, and 1∕*t* represents the reduction rate of channels. Moreover, to ensure the input channel dimension is consistent with the output channel dimensional, and the hyperparameter 1∕*r* (referring to the proportion of the input channel of the sub-branch output channel) is adopted in this paper, here we empirically set *r* to 6.

**Table 1:** Data processing in the Shield-block.

Input dimension	Operator	Output dimension
$ \left[ \begin{array}{@{}l@{}} \displaystyle H\times W\times C\\ \displaystyle H\times W\times CH\times W\times C\\ \displaystyle H\times W\times C\times \frac{1}{t} H\times W\times C\\ \displaystyle H\times W\times C\times \left( 1- \frac{1}{r} \right) \\ \displaystyle \hspace*{20.00003pt}\hspace*{20.00003pt}\hspace*{20.00003pt}\hspace*{10.00002pt}\text{} \end{array} \right] $	$ \left[ \begin{array}{@{}l@{}} \displaystyle 3\times 3\text{Dwise Conv,}\RE lu6\\ \displaystyle 1\times 1\text{Conv, Linear}3\times 3\text{Dwise Conv, Relu6}\\ \displaystyle 1\times 1\text{Conv, Re}lu61\times 1\text{Conv},\text{Re}lu6\\ \displaystyle 3\times 3\text{Dwise Conv, Linear}\\ \displaystyle \hspace*{20.00003pt}\hspace*{20.00003pt}\hspace*{20.00003pt}\hspace*{10.00002pt}\hspace*{10.00002pt}\hspace*{10.00002pt}\hspace*{10.00002pt}\hspace*{10.00002pt}\text{Concat} \end{array} \right] $	$ \left[ \begin{array}{@{}l@{}} \displaystyle H\times W\times C\\ \displaystyle H\times W\times C\times \frac{1}{t} H\times W\times C\\ \displaystyle H\times W\times C\times \left( 1- \frac{1}{r} \right) H\times W\times C\times \frac{1}{r} \\ \displaystyle H\times W\times C\times \left( 1- \frac{1}{r} \right) \\ \displaystyle \hspace*{10.00002pt}\hspace*{10.00002pt}\hspace*{10.00002pt}\hspace*{10.00002pt}\hspace*{10.00002pt}\hspace*{10.00002pt}\hspace*{10.00002pt}H\times W\times C \end{array} \right] $

**Figure 3:**
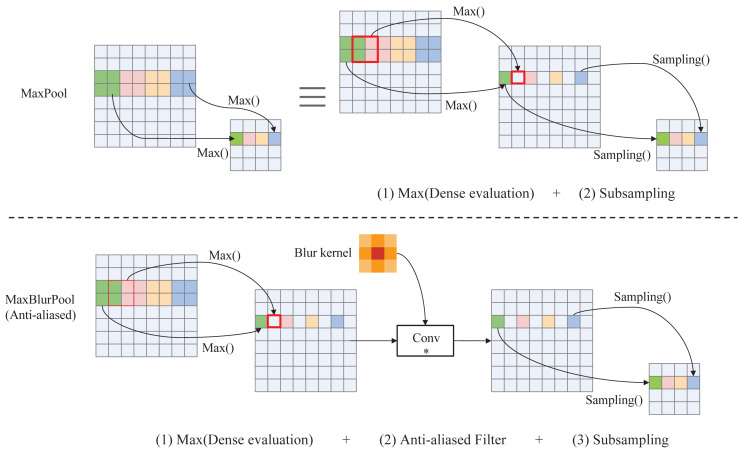
Operation details of MaxPool and anti-aliased MaxBlurPool.

***C.Down-sampling***. Down-sampling operator can reduce the feature dimensionality while retaining the valid information. Traditional down-sampling methods (eg. MaxPool, Strided-Convolution, AvgPool, etc.) violate the shift-equivariance and results in small shifts in the input that can drastically change the output (Azulay & Weiss, 2018). And this phenomenon will become more obvious with the increase of the network depth. To solve this problem, a fuzzy sampling method, namely MaxBlurPool, proposed in (Zhang, 2019) is adopted in our method. The specific process of MaxPool and MaxBlurPool is shown in Fig. 3. From this figure, we can see that a blur kernel is inserted between max and subsampling to remove aliased in the MaxBlurPool method, thereby improving the shift-invariant and enhancing the robustness of the CNN model.

### Lightweight sub-feature space attention module (SFSAM)

**Figure 4:**
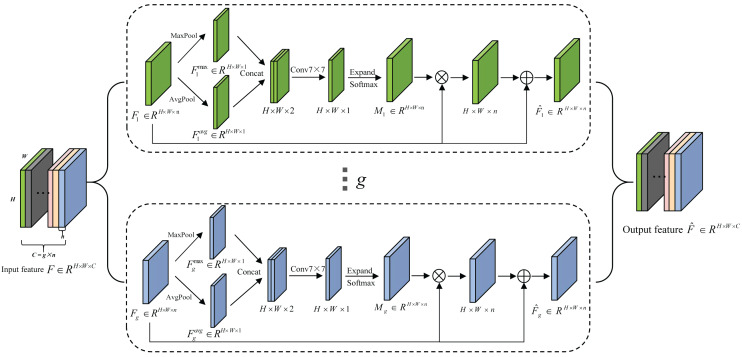
The schematic diagram of SFSAM structure.

Due to the low discrimination of different types of seeds, the fine-grained spatial features are crucial for seed sorting. Therefore, an extremely lightweight sub-feature space attention module is proposed to selectively emphasize fine-grained features, and suppress the interference of complex backgrounds. It divides the feature maps into different subspaces and infers different attention maps for each subspace, thus can generate the multi-scale feature representation, it is shown in Fig. 4. The detailed process is described as follows.

The input feature map *F* ∈ *R*^*H*×*W*×*C*^ is firstly divided into *g* mutually exclusive groups $ \left[ {F}_{1},{F}_{2},{F}_{3},\ldots ,{F}_{i},\ldots ,{F}_{g} \right] $ (i.e., sub-feature spaces),where each sub-feature space *F*_*i*_ contains *n* intermediate feature maps. Zagoruyko & Komodakis (2016) have proved that pooling operations along the channel axis are effective in highlighting informative regions. Therefore, AvgPool and MaxPool operations are applied to *g* sub-feature spaces along the channel axis to generate *g* groups of average-pooled features ${F}_{i}^{\max }\in {R}^{1\times H\times W}$ and max-pooled features ${F}_{i}^{avg}\in {R}^{1\times H\times W}$. Then, these features are concatenated separately to generate *g* efficient feature descriptors $ \left[ {F}_{i}^{\max },{F}_{i}^{avi} \right] $. Thereafter, the *g* group’s subspace attention maps are generated using Eq.(1).

(1) \begin{eqnarray*}{M}_{i}={soft}\nolimits \max \nolimits ({f}^{k\times k}([{MaxPool}\nolimits \left( {F}_{i} \right) ,{AvgPool}\nolimits ({F}_{i})]))\nonumber\\\displaystyle ={soft}\nolimits \max \nolimits \left( {f}^{k\times k} \left( \left[ {F}_{i}^{\max \nolimits },{F}_{i}^{avi} \right] \right) \right) \end{eqnarray*}

where *f*^*k*×*k*^ represents a convolution operation with a filter size of *k* × *k*. In this paper, *k* is empirically set to 7. The attention map in each group (subspace) can capture the non-linear dependencies among the feature maps by learning to gather cross-channel information. Meantime, we employ a gating mechanism with a softmax activation to map the attention weighting tensor into [0, 1].

Then, each group of feature maps gets the refined set of feature maps (${\hat {F}}_{i}$) after the feature redistribution in Eq. (2).

(2) \begin{eqnarray*}{\hat {F}}_{i}= \left( {M}_{i}\otimes {F}_{i} \right) \oplus {F}_{i}\end{eqnarray*}

where ⊗ is element-wise multiplication and ⊕ is element-wise addition.

The final output $\hat {F}$ of SFSAM is obtained by concatenating the feature maps of each group, and it is described as Eq. (3).

(3) \begin{eqnarray*}\hat {F}={concat}\nolimits \left( {\hat {F}}_{1},{\hat {F}}_{2},{\hat {F}}_{3},\ldots ,{\hat {F}}_{i},\ldots ,{\hat {F}}_{g} \right) .\end{eqnarray*}

### Network topology

In this paper, a novel lightweight CNN model with a dual-branch network structure based on visual attention, denoted as SeedSortNet, is proposed for seed sorting with higher efficiency and recognition accuracy. The setting of the proposed SeedSortNet is outlined in Table 2. Each row denotes a sequence of building blocks, which is the repeated times of ‘R’. The reduction ratio of channels is used in each Shield-block is denoted by ‘ 1∕*t*’, and ‘C’ represents the number of channels in the output feature map.

**Table 2:** Parameter configuration diagram of the SeedSortNet.

Stage	Input	Operator	*t*	C	R
1	224×224×3	Root-module	—	32	1
2	112×112×32	Shield-block	2 |6	64	2
3	112×112×64	SFSAM	—	64	1
4	112×112×64	MaxBlurPool	—	64	1
5	56×56×64	Shield-block	2 |6	128	4
6	56×56×128	SFSAM	—	128	1
7	56×56×128	MaxBlurPool	—	128	1
8	28×28×128	Shield-block	2 |6	128	5
9	28×28×128	SFSAM	—	192	1
10	28×28×192	MaxBlurPool	—	192	1
11	14×14×192	Shield-block	2 |6	256	4
12	14×14×256	SFSAM	—	256	1
13	14×14×256	MaxBlurPool	—	256	1
14	7×7×256	GlobalAvgpool	—	256	—
15	1×1×256	Dropout 2D-FC	—	2	—

We first use Root-module to generate 32 feature maps with the size of 112×112. Then, it is followed by the 15 Shield-blocks, four SFSAM attention modules, and four down-sampling layers (i.e., MaxBlurPool) spatial location distributions described in Table 2. At the first Shield-block of stages 2, 5, 8, and 11, the identity mappings do not need to be set because of the increasement of the feature map depth. Besides, we set ‘*t*’ to 2 to avoid the information loss due to the low-dimensional input. Finally, the output of the fourth down-sampling layer is followed by a global average pooling layer, which can convert 2D feature maps into 1D feature vectors.

## Experiments

### Experimental datasets

In this section, two datasets are selected for verifying the effectiveness of the proposed network architecture.

#### Maize seed dataset

The first dataset is a public haploid and diploid maize seed dataset of the maize research institute in Sakarya (Turkey), including 3000 RGB images of corn seeds (Altunta, Cömert & Kocamaz, 2019), and it includes 1230 haploid seeds images and 1770 diploid seed images. The dimensions of these images depend on the sizes of the seeds and vary between 300×289 pixels and 610×637 pixels. In the experiment, three-quarters of the dataset are used for training, and the remaining images are used for testing, as shown in Table 3.

The number of maize seed dataset is limited and may bring the overfitting for the proposed model. Therefore, the data augmentation methods, such as horizontal flip, vertical flip, and angle rotation, are adopted to augment the maize seed data set by a factor of 4. The experimental results prove that such a large dataset is enough to train a model with very strong generalization ability.

#### Sunflower seed dataset

**Figure 5:**
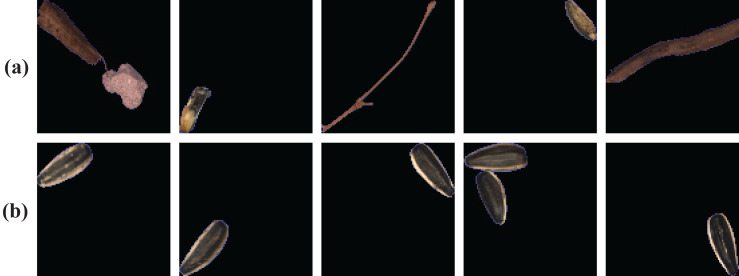
Representative samples of sunflower seed dataset. (A) Abnormal sunflower seeds; (B) normal sunflower seeds.

To thoroughly evaluate the effectiveness of the proposed method, we constructed our sunflower seed dataset on an industrial production line for the experiments. The image acquisition device equipped with a color line scan camera is established to collect 15834 sunflower seed RGB images with the size of 100×100 pixels. And we divided them into two categories, as shown in Fig. 5. The top row is the abnormal seed images composed of leaves, stones, defective seeds, etc. The bottom row is the normal sunflower seed images. It is worth noting that when the picture contains several seeds and impurities or hybrids, we will classify them as abnormal to ensure a low false alarm. In our experiment, about three-quarters of the dataset are randomly selected as the training set, and the remaining images are used for testing, as shown in Table 3.

**Table 3:** Category distribution and proportion of the training set on maize seed dataset and sunflower seed dataset.

Dataset	Category distribution	The proportion of the training set
Maize	1770 (Diploid) 1230 (Haploid)	75%
Sunflower	7837 (Normal) 7997 (Abnormal)	≈75%

### Implementation details and evaluation metric

#### Implementation details

All experiments were performed on a 64-bit Linux-based operation system, Ubuntu 18.04. The software is mainly based on the deep learning architecture of Pytorch and python development environment Spyder. The hardware is based on an Intel(R) Xeon(R) CPU E5-2650 v4 @2.20 GHz and two NVIDIA Quadro M5000 GPUs, with CUDA10.2 accelerating calculation.

And we train the network by mini-batch SGD, with an initial learning rate of 0.001 and a reducing factor of 0.1 after 30 epochs. The momentum parameter is set to 0.9 and the weight decay parameter is 0.0001. The number of iterations in training is 100, and the batch size is set to 16 and 64 on the maize and sunflower datasets, respectively. Besides, the input image size is resized to 224×224-pixel by the CenterCrop function, and the parameter *g* is set to 4 by analyzing experimental results.

#### Evaluation metric

To quantitively evaluate the effectiveness of the proposed method, four metrics, such as true positive (*tp*), true negative (*tn*), false positive (*fp*), and false negative (*fn*) are adopted in our method. *tp* is the true positive and represents correctly recognized haploid maize seeds or the normal sunflower seed. *tn* is the true negative and represents correctly recognized diploid maize seeds or the abnormal sunflower seed. *fp* is the false positive and represents the falsely recognized haploid maize seeds or the normal sunflower seed. *fn* is the false negative and represents falsely recognized diploid maize seeds or the abnormal sunflower seed. Based on these metrics, four evaluation metrics, accuracy (*Acc*), precision (*p*), recall (*r*) and F1-score, are calculated as Table 4.

**Table 4:** Calculation formulas and explanations of binary class metrics.

Measure	Formulation	Evaluation Focus
Accuracy (*Acc*)	$ \frac{tp+tn}{tp+fp+tn+fn} $	The overall accuracy of a model.
Precision (*p*)	$ \frac{tp}{tp+fp} $	The ratio of correctly classified positive samples to estimated total positive sample.
Recall (*r*)	$ \frac{tp}{tp+fn} $	The proportion of positive values classified as true.
F1-score	$ \frac{2\ast p\ast r}{p+r} = \frac{2\ast \frac{tp}{tp+fp} \ast \frac{tp}{tp+fn} }{ \frac{tp}{tp+fp} + \frac{tp}{tp+fn} } $	The harmonic mean between precision and recall.

It should be noted that the F1-score metric can better interpret the true performance when the number of samples is not balanced. Receiver operating characteristic (ROC) curves are also a useful tool for measuring a model performance without considering class distribution or error costs. Also, the number of parameters and required float points operations (denoted as FLOPs) are also employed to evaluate the model size and computational complexity, which are widely-used protocols.

### Result analysis

#### Results on maize seed dataset

To assess the performance of our network (i.e., SeedSortNet) in the maize dataset. Six representative CNN models (i.e., AlexNet, VGG, ResNet, GoogleNet, DenseNet (Huang et al., 2017), and Resnext (Xie et al., 2017) are selected to conduct comparative experiments. The experimental results are shown in Table 5.

**Table 5:** Performance comparison of different network on maize seed dataset.

Model	Parameters	FLOPs	Acc	Precision	Recall	F1-score
AlexNet	57.01M	711.46M	93.33	93.39	92.80	93.07
VGG11	128.77M	7.63G	94.67	94.54	94.43	94.48
VGG13	128.96M	11.33G	94.50	94.51	94.10	94.29
ResNet18	11.18M	1.82G	95.33	95.18	95.18	95.18
ResNet50	23.51M	4.12G	96.00	95.73	96.05	95.88
DenseNet121	6.96M	2.88G	95.83	95.58	95.85	95.71
GoogleNet	5.60M	1.51G	96.67	96.50	96.62	96.56
ResNext101	86.75M	16.48G	96.00	95.77	95.99	95.88
SeedSortNet	0.40M	512.06M	97.33	97.30	97.18	97.24

From the results in Table 5, we can observe that the adopted network can achieve good classification accuracy, and reach more than 90% under the same experimental environment. SeedSortNet has the best performance, with accuracy, precision, recall, and F1-score of 97.33%, 97.30%, 97.18%, and 97.24%, respectively, with a relatively low computational complexity and model size. These results verify the effectiveness of the proposed method.

Meanwhile, we also compared the performance of mainstream lightweight CNN models (eg, MobileNetv1, MobileNetv2, ShuffleNetv1, ShuffleNetv2, GhostNet) under different calculation benchmarks. The experimental results on the maize dataset in terms of computational complexity, model parameters, classification accuracy, and F1-score are shown in Table 6. The models are typically grouped into two levels of computational complexity for embedded device applications, i.e., ∼300MFLOPs and 500 ∼600MFLOPs. From the results, we can see that the larger FLOPs lead to higher accuracy in these lightweight networks. SeedSortNet outperforms other competitors consistently in classification accuracy and F1-score at various computational complexity levels. Furthermore, the number of parameters has also greatly decreased for the proposed method.

**Table 6:** Performance comparison of maize seed dataset in lightweight CNNs.

Model	Parameters	FLOPs	Acc	F1-score
MobileNetv1	3.22M	587.94M	94.00	93.81
MobileNetv2 1.4×	4.06M	566.33M	96.00	95.89
ShuffleNetv1 2×(*g*=3)	3.53M	537.48M	96.67	96.55
ShuuffleNetv2 2×	5.35M	591.79M	96.00	95.90
GhostNet 2×	12.96M	529.89M	96.50	96.41
SeedSortNet	0.40M	512.06M	97.33	97.24
MobileNetv1 0.75×	1.83M	339.80M	91.83	91.65
MobileNetv2	2.23M	318.96M	95.83	95.71
ShuffleNetv1 1.5×(*g*=3)	2.00M	301.90M	96.17	96.05
ShuffleNetv2 1.5×	2.48M	302.65M	95.50	95.38
GhostNet 1.5×	7.79M	310.76M	95.83	95.72
SeedSortNet 0.75×	0.23M	338.64M	97.00	96.90

**Figure 6:**
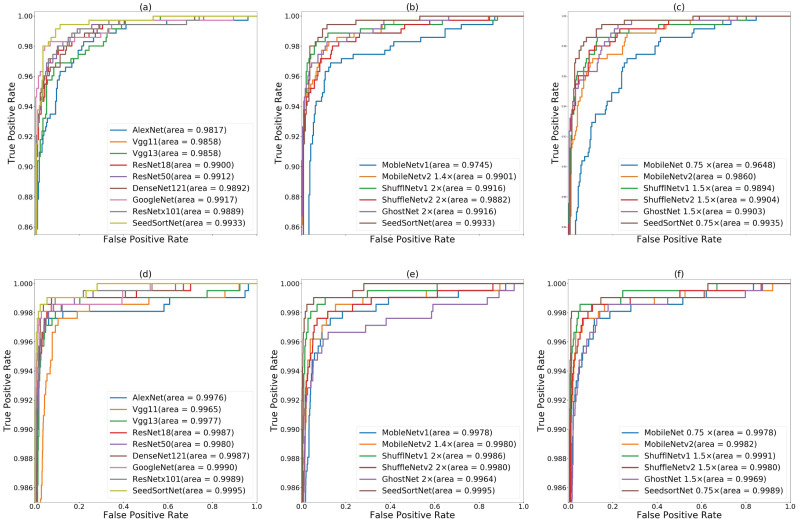
ROC curves of the CNN models on maize seed dataset (A, B, C) and sunflower seed dataset (D, E, F). (A & D) ROC curves of SeedSortNet and Six representative CNN models (i.e., AlexNet, VGG, ResNet, GoogleNet, DenseNet, and Resnext), (B & E) ROC curves of the lightweight network (500 ∼600MFLOPs), (C & F) ROC curves of the lightweight network (∼300MFLOPs).

In order to further demonstrate the effectiveness of the proposed method, ROC curve is adopted to measure the model performance. Figures 6A–6C shows the ROC curves and the calculated area under curve (AUC) scores for using the proposed method and other network models (i.e., the above comparison network) on the maize seed dataset. From AUC scores, it is observed that the method achieve the best result of 99.33% compared with other models, which is superior to the above representative representative CNN models and lightweight networks.

#### Results on sunflower seed dataset

Table 7 demonstrates the model size, computational complexity, accuracy, recall, specificity and F1-score of different network models on the sunflower seed dataset. From the table, we can see that the proposed method has the highest accuracy, precision, recall, and F1-score, while it has the lower FLOPs and parameters.

**Table 7:** Performance comparison of different network on sunflower seed dataset.

Model	Parameters	FLOPs	Acc	Precision	Recall	F1-score
AlexNet	57.01M	711.46M	99.00	99.01	98.99	99.00
VGG11	128.77M	7.63G	97.78	97.78	97.80	97.78
VGG13	128.96M	11.33G	98.44	98.43	98.45	98.44
ResNet18	11.18M	1.82G	99.05	99.04	99.05	99.05
ResNet50	23.51M	4.12G	99.22	99.22	99.22	99.22
DenseNet121	6.96M	2.88G	98.90	98.89	98.91	98.90
GoogleNet	5.60M	1.51G	99.34	99.34	99.34	99.34
ResNext101	86.75M	16.48G	99.22	99.22	99.22	99.22
SeedSortNet	0.40M	512.06M	99.56	99.56	99.56	99.56

Similar to the maize seed sorting, we also conducted comparative experiments with the mainstream lightweight CNN models (eg, MobileNetv1, MobileNetv2, ShuffleNetv1, ShuffleNetv2, GhostNet) under different calculation benchmarks on the sunflower seed dataset. From Table 8, we can find that the classification accuracy and F1-score of the SeedSortNet are higher than other network models under a similar calculation cost. Meantime, we find that the test dataset is relatively balanced, thus its F1-score and accuracy are almost the same. Therefore, the proposed SeedSortNet is more suitable for deployment on edge devices and has the ideal sorting accuracy.

**Table 8:** Performance comparison of sunflower seed dataset in lightweight CNNs.

Model	Parameters	FLOPs	Acc	F1-score
MobileNetv1	3.22M	587.94M	98.36	98.36
MobileNetv2 1.4×	4.06M	566.33M	98.83	98.83
ShuffleNetv1 2×(*g*=3)	3.53M	537.48M	99.19	99.19
ShuuffleNetv2 2×	5.35M	591.79M	99.00	99.00
GhostNet 2×	12.96M	529.89M	98.80	98.80
SeedSortNet	0.40M	512.06M	99.56	99.56
MobileNetv1 0.75×	1.83M	339.80M	98.32	98.31
MobileNetv2	2.23M	318.96M	98.90	98.90
ShuffleNetv1 1.5×(*g*=3)	2.00M	301.90M	99.12	99.12
ShuffleNetv2 1.5×	2.48M	302.65M	98.73	98.73
GhostNet 1.5×	7.79M	310.76M	98.44	98.44
SeedSortNet 0.75×	0.23M	338.64M	99.34	99.34

In Figs. 6D–6F, we find that the AUC score of the proposed method is closer to 1.0 (i.e., 0.9995) on the sunflower seed dataset compared with other CNN models, which demonstrates SeedSortNet has a good ability to prevent misclassification.

### Ablation study

The ablation study is carried on SeedSortNet and the network without SFSAM attention mechanism. The experimental results in Table 9 show that F1-score of 96.33% and 99.37% are obtained without SFSAM on the maize and sunflower seed datasets, respectively, which proves that Root-model and Shield block have better information extraction abilities. Meanwhile, SeedSortNet can get 97.33% and 99.56% F1-score, respectively. These demonstrate that SFSAM can selectively emphasize information features and suppress the interference of complex backgrounds, thereby improving the performance. At the same time, it can also be observed from Table 9 that SFSAM does not introduce too many parameters and calculations.

**Table 9:** F1-score of SeedSortNet and SeedSortNet (without SFSAM) on maize seed dataset and sunflower seed dataset.

Model	Parameters (M)	FLOPs	F1-score(maize)	F1-score (sunflower)
SeedSortNet(without SFSAM)	0.399M	505.26M	96.22	99.39
SortSeedNet	0.400M	512.06M	97.24	99.56

### Effects of g selection in SFSAM

As described in the SFSAM section, the feature maps are divided into *g* groups and generate *g* attention maps. Each attention map can capture cross-channel information from the feature maps in its respective group. When *g* = 1, the cross channel information for the whole feature volume is captured by a single attention map, which is not sufficient to capture the complex relationships in the entire feature space and will result in lower predictive performance. When 1 < *g* < *C*, the better exchange of cross-channel information can be obtained. Therefore, we conduct experiments on the different parameters assigned by *g* (such as *g* = 1, 4, 8, 16), and the results in Table 10 confirm the correctness of the above analysis. It can also be observed that the maize and sunflower seed datasets have achieved higher performance gains, and the FLOPs and parameters increase with the increase *g*. Based on the experimental results, we adopt *g* = 4 to conduct the above series of comparative experiments which provides a reasonable trade-off between preserving good performance and improving computational efficiency.

**Table 10:** F1-score of SeedSortNet (with fewer parameters/FLOPs; *g* = 1, 4, 8, 16) on maize seed dataset and sunflower seed dataset.

Model	Parameters	FLOPs	F1-score(maize)	F1-score(sunflower)
SortSeedNet (*g*=1)	0.399M	506.56M	96.39	99.44
SortSeedNet (*g*=4)	0.400M	512.06M	97.24	99.56
SortSeedNet (*g*=8)	0.402M	518.85M	96.90	99.51
SortSeedNet (*g*=16)	0.405M	532.45M	96.57	99.46

## Discussion

In this paper, we present a rapid and highly efficient lightweight CNN for seed sorting (i.e., SeedSortNet). We first design a novel dual-branch lightweight feature extraction module (i.e., Shield block) for building efficient neural network architectures. In the down-sampling layer, MaxBlurPool is employed instead of frequently-used MaxPool to improve the shift-invariant of the network. Then we proposed a lightweight sub-feature space attention module (SFSAM), which improves the representational power of the model by learning different attention feature maps. A wide range of experiments show the effectiveness of SeedSortNet, which achieves state-of-the-art identification performance on maize seed and sunflower seed datasets while utilizing fewer parameters and lower computational complexity. In future research, the number of seed varieties and images will be further increased to test the performance of these models, and we hope that these methods can be applied in the seed market.

## Additional Information and Declarations

### Data Availability

The following information was supplied regarding data availability: The maize seed dataset comes from Altuntaş et al. (2019): https://doi.org/10.1016/j.compag.2019.104874 and is available at: http://www.rovile.org/datasets/haploid-and-diploid-maize-seeds-dataset/. The seedsortnet code and sunflower seed dataset are available at GitHub: https://github.com/Huanyu2019/Seedsortnet.
